# Vermessung und Vermittlung der Krise in den frühen Lageberichten zur Corona-Pandemie: ein deutsch-französischer Vergleich

**DOI:** 10.1007/s41244-021-00214-2

**Published:** 2021-09-29

**Authors:** Christian Koch, Britta Thörle

**Affiliations:** Romanisches Seminar, Universität Siegen, Siegen, Deutschland

**Keywords:** Covid-19, Pressekonferenz, Krisenkommunikation, Experten-Laien-Kommunikation, Argumentationsmuster, Nomination, Covid-19, Press Conference, Crisis Communication, Expert Lay Communication, Argumentation Patterns, Nomination

## Abstract

Mit Ankunft des Coronavirus in Europa im Frühjahr 2020 beginnen in Deutschland und Frankreich relativ zeitgleich Lageberichte durch die staatlichen Gesundheitsinstitutionen – das Robert Koch-Institut und die Direction générale de la santé. Diese mündlich gehaltenen Expertenvorträge dienen sowohl zur Beschreibung der aktuellen Verbreitung des Virus als auch zur Begründung der zu ergreifenden Maßnahmen. Untersucht wird in diesem Artikel eine Reihe von Lageberichten beider Länder im Hinblick auf Ähnlichkeiten und Unterschiede auf sprachlicher Ebene. Neben den verschiedenen kommunikativen Handlungen des Beschreibens und Interpretierens der Sachlage, des Argumentierens für Maßnahmen sowie des Empfehlens und Anordnens von Handlungen wird die neu entstehende Lexik zur Bezeichnung des Virus und der Masken genauer betrachtet. Die Gegenüberstellung von deutschen und französischen Textauszügen zeigt, dass die beiden parallel, aber voneinander unabhängig stattfindenden Veranstaltungen zahlreiche Ähnlichkeiten aufweisen. Unterschiede, die gezeigt werden können, betreffen im Bereich der Maßnahmen und Appelle die divergierende politische Funktion der beiden Einrichtungen mit einer stärker beratenden Rolle des Robert Koch-Instituts gegenüber der Direction générale de la santé als Teil des Gesundheitsministeriums und damit als dem stärkeren politischen Entscheidungsträger.

## Abstract (en)

With the arrival of the coronavirus in Europe in early 2020, situation reports by the state health institutions – the Robert Koch-Institut and the Direction générale de la santé – begin in Germany and France at relatively the same time. These oral presentations by experts serve both to describe the current spread of the virus and to justify the measures that need to be taken. This article examines a number of situation reports from both countries with regard to similarities and differences at the linguistic level. In addition to the communicative acts of describing and interpreting the situation, arguing for measures, and giving recommendations and orders, the newly emerging lexis for naming the virus and the masks will be examined. The comparison of German and French text excerpts shows that the two events, which take place in parallel but independently from each other, have numerous similarities. Differences that can be shown concern, in the field of measures and appeals, the diverging political function of the two health institutes, with a rather advisory role of the Robert Koch-Institut compared to the Direction générale de la santé as part of the Ministry of Health and thus as the stronger political decision-maker.

## Einleitung

Seit Ausbruch der Covid-19-Pandemie ist es in einigen Ländern zur Routine geworden, dass Wissenschaftler:innen im Auftrag der Regierungen vor die Öffentlichkeit treten, um über Infektionszahlen, Todesfälle und Maßnahmen im Zusammenhang mit Covid-19 zu informieren und die jeweils aktuelle nationale Lage zu bewerten. Dies geschieht u.a. in regelmäßigen und zeitweise täglichen Pressekonferenzen von Ministerien oder staatlichen Institutionen, die durch ihre Verbreitung über Social-Media-Plattformen und TV-Kanäle sowie dadurch, dass z.B. Nachrichtensendungen auf sie Bezug nehmen, einem breiten Publikum bekannt und zugänglich sind. Die beteiligten Wissenschaftler:innen, in der Regel Virolog:innen und Epidemiolog:innen, erhalten damit vor allem ab Februar/März 2020 ein bis dahin nicht gekanntes Ausmaß an öffentlicher Aufmerksamkeit und werden zu zentralen Akteur:innen im medialen und politischen Diskurs, in dem sich von da an vieles um Messzahlen, Modellrechnungen und Prognosen dreht.

In diesem Beitrag beschäftigen wir uns mit den nur wenige Wochen nach Ausbruch der Covid-19-Pandemie einsetzenden Pressekonferenzen des deutschen Robert Koch-Instituts (RKI) und der Direction générale de la santé (DGS) im französischen Gesundheitsministerium. In beiden Fällen handelt es sich um wissenschaftliche Einrichtungen, deren staatlicher Auftrag der Gesundheits- und insbesondere der Infektionsschutz ist und denen deshalb eine wichtige Rolle in der Pandemiebekämpfung zufällt. Die Pressekonferenzen sind vor diesem Hintergrund auch als Organe der staatlichen Krisenkommunikation zu betrachten.^1^ In der Analyse geht es uns zum einen um die Frage, wie die (Krisen‑)Situation von den Beteiligten auf der Sachverhalts‑, Argumentations- und Handlungsebene kommunikativ konstituiert und vermittelt wird und welchen Beitrag die Pressekonferenzen zur öffentlichen Verhandlung der Krise leisten (Abschnitt 3). Ein besonderer Fokus liegt dabei in einem weiteren Schritt auf der Lexik und der Verhandlung von Nominationen zum Ausdruck neuartiger Sachverhalte (Abschnitt 4). Das Interesse am Vergleich der Lageberichte zweier Länder liegt unseres Erachtens darin, dass hier zwei voneinander unabhängige Formate entstanden sind, die aufgrund der relativ zeitgleichen Wahrnehmung^2^ der akut werdenden Gesundheitskrise in beiden Ländern große Gemeinsamkeiten aufweisen. Zu diesem Zweck konzentriert sich die Analyse auf den Zeitraum von Mitte bis Ende März 2020, in dem in beiden Ländern erstmals weitgehende Ausgangs- und Kontaktbeschränkungen in Kraft treten.

## Korpus: Pressebriefings des RKI und *Points de situation* der DGS

Das Robert Koch-Institut (RKI) ist ein Bundesinstitut im Geschäftsbereich des Bundesministeriums für Gesundheit und die zentrale Einrichtung der Bundesregierung auf dem Gebiet der Gesundheitsüberwachung und -prävention. Die Kernaufgaben des RKI sind die Erkennung, Verhütung und Bekämpfung von Krankheiten, insbesondere von Infektionskrankheiten.^3^ Das erste Pressebriefing des RKI zu Covid-19 findet am 27.02.2020 statt. Es folgen von da an regelmäßige – im Untersuchungszeitraum nahezu tägliche – Pressekonferenzen, die im TV-Programm Phoenix ausgestrahlt werden und gleichzeitig auf YouTube abrufbar sind. Die Pressebriefings werden hauptsächlich vom Präsidenten des RKI, Lothar Wieler, und gelegentlich auch von seinem Stellvertreter Lars Schaade durchgeführt.

Die *Points de situation* werden vom französischen Gesundheitsministerium, genauer von der Direction générale de la santé (DGS), einer Sektion des Ministeriums, verantwortet, deren Aufgabenbereich etwas weiter gefasst ist als der des RKI. Die DGS ist u.a. für den Gesundheitsschutz der Bevölkerung und die Organisation des Gesundheitswesens zuständig. Der erste *Point de situation* wurde von der Gesundheitsministerin Agnès Buzyn am 21.01.2020 durchgeführt. Nach ihrem Rücktritt im Februar 2020 sind ihr Nachfolger Olivier Véran sowie die Premierminister Édouard Philippe und später Jean Castex in einigen *Points de situation* vertreten. Die meisten Pressekonferenzen werden jedoch vom Leiter der DGS, Jérôme Salomon, durchgeführt, der entweder allein oder gemeinsam mit den Regierungsvertreter:innen auftritt. Wie beim RKI finden die *Points de situation* in dem von uns untersuchten Zeitraum ebenfalls fast täglich statt.

Für die Untersuchung wurden auf den Seiten des französischen Gesundheitsministeriums bzw. auf Phoenix und YouTube veröffentlichte Mitschnitte der Pressekonferenzen mithilfe eines Spracherkennungsprogramms automatisch transkribiert, für die Analyse relevante Stellen korrigiert und in ein Basistranskript^4^ umgewandelt. In der folgenden Darstellung beziehen wir uns auf den Zeitraum von Mitte März bis Anfang April 2020, wobei die Pressekonferenzen eines Tages, nämlich des 17.03.2020, zur direkteren Vergleichbarkeit im Vordergrund stehen. In Frankreich gehen diesem Datum die Einführung strenger Ausgangs- und Kontaktbeschränkungen am 14.03.2020 sowie die viel beachtete Rede Emmanuel Macrons am 16.03.2020 voraus. In Deutschland gibt es zu diesem Zeitpunkt bereits erste Kita- und Schulschließungen. Ausgangs- und Kontaktbeschränkungen werden erst ab dem 22.03.2020 eingeführt. Auf weitere Pressekonferenzen werden wir an passender Stelle punktuell Bezug nehmen.^5^

## »eine tägliche lageeinschätzung das ist unser job« (RKI, 03.04.20) – Die Lageberichte im Rahmen der Pressekonferenzen

Die Pressekonferenzen der DGS und des RKI sind komplexe kommunikative Ereignisse, die sich durch verschiedene Handlungsphasen und -schemata mit wechselnden Beteiligungskonstellationen (*participation frameworks* im Sinne von Goffman 1981) auszeichnen. So werden die Pressekonferenzen des RKI von der Pressesprecherin des Instituts moderiert, die die Anwesenden begrüßt, den Lagebericht ankündigt und das Rederecht in der darauffolgenden Fragerunde organisiert. Einige Pressekonferenzen der DGS beginnen mit der Ansprache des Gesundheitsministers oder anderen Regierungsvertreter:innen, an die sich der *Point de situation* und die Fragerunde der Presse anschließen. Der monologisch vorgetragene Situations- bzw. Lagebericht ist dabei als kommunikativ klar abgegrenzte Gesprächsphase innerhalb der Pressekonferenzen erkennbar, die von den Vortragenden etwa durch Begrüßungen und Adressierungen am Anfang sowie Dankesformeln am Ende rituell gerahmt werden und auf die metakommunikativ Bezug genommen werden kann:

1. RKI, 13.03.20, 00:16-00:28

((Susanne Glasmacher, Pressesprecherin:))01 heute is:t (.)äh professor lothar wieler (.)02 ähm ihr gesprächspartner, (.)03 der präsident des robert koch-instituts, (.)04 °h der wie üblich (.)05 äh **ein:e kurze einschätzung der lage** geben wird, (.)06 °h und danach für ihre fragen zur verfügung steht […]

(2) DGS, 30.04.20, 00:19-00:44

((Olivier Véran, Gesundheitsminister:))01 je vais vous présenter pour commencer ce point-presse,   *ich werde Ihnen zu Beginn dieses Pressebriefings*02 °hh les cartes (-)   *die Karten vorstellen*03 qui sont amenée:s (.) à nous guider dans les choix, (--)   *die dafür da sind uns bei den Entscheidungen zu leiten*04 en vue de la levée progressive du confinement, (-)   *im Hinblick auf die schrittweise Aufhebung des Lockdowns*05 tel qu’annoncé (.) jusqu’au (.) à partir du 11 mai.   *wie angekündigt bis zum ab dem 11. Mai*06 °h **ensuite je laisserai le directeur général de la santé**      *dann werde ich dem Generaldirektor für Gesundheit*07 **le professeur **(--) **jérôme salomon**,   *Professor Jérôme Salomon*08 **faire** (.) **le point** (.) **quotidien**   *den täglichen Lagebericht überlassen*09 tel que vous avez pris l’habitude,   *wie Sie es gewohnt sind*10 et puis il répondra (-) aux éventuelles questions (.)   *und danach wird er antworten auf eventuelle Fragen*11 des journalistes. (--)   *der Journalisten*


In der folgenden Betrachtung konzentrieren wir uns auf diesen Teil der Pressekonferenzen, wobei wir der Frage nachgehen, wie die Lage von den Akteuren kommunikativ vermittelt wird. Auf welche Sachverhalte wird Bezug genommen? Welche Relationen werden zwischen diesen hergestellt? In welche Argumentation wird die Darstellung eingebunden? Welche kommunikativen Zwecke lassen sich daraus ableiten? Wie wird das Pandemiegeschehen sprachlich als Krise konstituiert?

### Die »daten zur lage« (RKI, 17.03.20) – Fallzahlen und ihre Ordnung

Das dominante und über alle Pressekonferenzen des Korpus hinweg konstante Element der Lageberichte ist das Verlesen aktueller Infektionszahlen sowie weiterer Daten, u.a. zu Sterbefällen und Hospitalisierungen. In der Regel beginnen die Lageberichte mit diesen Meldungen. Wie sehr sich die Pressekonferenzen in diesem Punkt ähneln, zeigen zwei Ausschnitte vom 17.03.2020:

(3) DGS, 17.03.20, 00:29-00:54

01 en ce qui concerne la situation internationale (.)   *im Hinblick auf die internationale Situation*02 de la pandémie, (.)   *der Pandemie*03 °h covid-19,      *Covid-19*04 nous sommes ce soir à (--) 190000 CAS, (-)   *sind wir heute Abend bei 190000 Fällen*05 80000 guéris, (1.2)   *80000 Genesenen*06 on peut noter que l’essentiel des cas est désormais (--)   *man kann anmerken, dass der wesentliche Anteil der Fälle jetzt*07 hors de chine,   *außerhalb von China vorliegt*08 puisque il y a 109000 cas hors de chine (-)   *da es 109000 Fälle außerhalb Chinas gibt*09 contre 81000 en chine   *gegenüber 81000 in China*10 où l’épidémie est quasi à l’arrêt, (-)   *wo die Epidemie fast zum Stillstand gekommen ist*11 °hh 155 pays (.) sont touchés, (---)       *155 Länder sind betroffen*

(4) RKI, 17.03.20, 00:41-01:19

01 ähm (.) schauen wir uns: (.)02 zunächst mal die situation in der welt an. (1.1)03 wir haben (--) momentan-04 also stand äh (-) gestern um 15 uhr, (.)05 °hh waren in china insgesamt 41/ (.)06 81000 fälle gemeldet. (1.6)07 ähm (-) und wir haben international inzwischen (--)08 MEHR fälle gemeldet (-)09 außerhalb chinas als IN china, (--)10 und zwar waren das gestern um 15 uhr (.)11 offiziell gemeldet (.)12 sechsundachtzigtausendsechshundert (-)13 einundsechzig fälle. (-)14 das betrifft 146 länder, (--)15 das zeigt also (das/dass) äh (-)16 was wir hier wissen, (.)17 es handelt sich um eine pandemie, (-)18 es handelt sich um ein virus19 das sich weltweit verbreitet, (--)


Mit Thema-einleitenden Routineformeln (»en ce qui concerne«, »schauen wir uns: (.) zunächst mal [...] an.«) beziehen die Sprecher sich zunächst auf die weltweite Situation. Diese wird durch die Nennung von Fallzahlen charakterisiert, wobei in beiden Beispielen zwischen China, das bis Anfang 2020 das Zentrum des epidemiologischen Geschehens war, und der Welt außerhalb Chinas unterschieden wird.^6^ Im weiteren Verlauf wird diese Art der Situationsbeschreibung für weitere Länder sowie verschiedene Regionen des jeweils eigenen Landes fortgesetzt:

(5) DGS, 17.03.20, 00:55-01:20

01 l’iran avec 16000 ca:s,   *der Iran mit 16000 Fällen*02 la corée du sud avec 8320, (.)   *Südkorea mit 8320*03 °h mais ce qui préoccupe évidemment,      *aber was offenkundig Sorge bereitet*04 c’est que l’euROPE (-)   *ist, dass Europa*05 °h est l’épicentre (.) de la pandémie, (.)      *das Epizentrum der Pandemie ist*06 avec plus de 65000 cas (-) et près de 3000 morts, (--)   *mit über 65000 Fällen und fast 3000 Toten*07 le pays le plus touché en nombre absolu (.)   *das in absoluten Zahlen am stärksten betroffene Land*08 après la chine (.)   *nach China*09 est l’italie, (--)   *ist Italien*10 avec 28000 cas; (---)   *mit 28000 Fällen*11 suivent l’espagne avec 11200 cas,   *gefolgt von Spanien mit 11200 Fällen*12 et l’allemagne avec 8600 cas. (---)   *und Deutschland mit 8600 Fällen*

(6) RKI, 17.03.20, 01:30-02:14

01 in italien, (--)02 gibt es bislang 24747 (.) offiziell gemeldete fälle, (-)03 °hhh und von diesen f/ haben wir berichtet04 von insgesamt 1809 (.) todesfällen. (-)[…]05 in südkorea, (--)06 sind mit gestrigem zeitpunkt 15 Uhr07 8162 fälle gemeldet, (.)08 °hh das ist ‘ne zunahme von knapp (.)09 ZEHN prozent gegenüber vorgestern, (.)10 °hh und hier wurden 75 todesfälle gemeldet. (---)


Durch die listenförmige Abfolge von Referenzen auf ein Land (»l’iran«, »la corée du sud«, »in italien«, »in südkorea«) und die Zuordnung der jeweiligen Anzahl von Infektions- bzw. Todesfällen als Prädikation ergibt sich das Muster einer Bestandsaufnahme oder Inventarisierung, das als elementare Form der Beschreibung aufgefasst werden kann (vgl. Adam 2017, S. 79).^7^ Prominentes Strukturierungsprinzip sind politisch definierte Territorien. Mit der Gliederung der Beschreibung in nationale Territorien (im späteren Verlauf der Lageberichte auch Landesregionen) wird die bildschematische Metapher von Ländern als containern im Sinne Lakoffs und Johnsons (1980) aufgerufen, in denen das Virus enthalten ist: Es wird zwischen den Infektionszahlen »hors de chine« und »en chine« bzw. »außerhalb chinas« und »IN china« (vgl. Beispiele 3 und 4) unterschieden. Sie werden in Südkorea, in Italien, in Deutschland usw. erfasst.^8^ Unterhalb der Ordnungsebene der Staaten spielen in den oben zitierten Beispielen die Kategorien »Fälle«/»cas« und »Todesfälle«/»morts« eine Rolle. Weitere Kategorien sind u.a. Hospitalisierungen, Infizierte mit schweren Krankheitsverläufen oder Genesene. Durch die Selektion und Anordnung der Beschreibungskategorien wird das vermittelte Wissen über die Pandemie auf spezifische Art und Weise strukturiert. Bestimmte Wissensaspekte und Perspektivierungen werden für die Lagebeschreibung relevant gesetzt, während andere ausgeblendet bleiben (vgl. dazu die Kritik von Contzen/Griem 2020, S. 245, an der Form der Liste und dem ihr zugrunde liegenden, »für die Rezipient*innen unsichtbaren Verfahren der In- und Exklusion«).^9^

Bei den Angaben, die den Kategorien zugeordnet werden, handelt es sich um tendenziell hohe gerundete (DGS) oder ungerundete (RKI) Zahlen. In den Pressekonferenzen des RKI fällt dabei die häufige Verwendung des Attributs »offiziell gemeldet« auf (Ausschnitt 4, Z. 11, Ausschnitt 6, Z. 02; vgl. auch an anderer Stelle die metasprachliche Hervorhebung: »und wie gesagt nochmal (.) hier geht es um offiziell gemeldete fälle«, RKI, 17.03.20, 01:56-02:00). »Offiziell gemeldet« heißt, dass es sich um die von den Gesundheitsämtern über eine Software an das RKI übermittelte Zahlen handelt (vgl. RKI 13.03.20). Die »offiziellen« Meldedaten werden teilweise mit konkurrierenden Angaben anderer Institutionen verglichen (etwa mit denen der John-Hopkins-University, vgl. Beispiel 8), es werden »laborbestätigte [...] fälle« von »offiziell eingetragen[en]« (Beispiel 7) unterschieden sowie die »tatsächlichen erkrankungszahlen« von denen, die dem RKI »übermittelt werden« (Beispiel 9), so dass der Sprecher insgesamt viel Redezeit auf die Klarstellung der Relativität der präsentierten Daten verwendet:

(7) RKI, 13.03.20, 06:21-06:28

01 das heißt also (--)02 zwischen den (-) **laborbestätigten fällen**03 und den **fällen die eben äh** (.)04 **offiziell eingetragen wurden**05 gibt’s immer ‘ne kleine lücke.

(8) RKI, 17.03.20, 02:22-02:27

01 äh sie finden auf der website (.) des john hopkins02 zahlen über 7000,03 wie gesagt diese zahlen kennen wir, […]

(9) RKI, 17.03.20, 10:16-10:32

01 wir müssen aber davon ausgehen (-)02 dass die **tatsächlichen erkrankungszahlen** (-)03 deutlich höher sind als **die** (-)04 **die dem robert koch-institut übermittelt werden**, (-)05 °hh das liegt zum einen,06 an den testkapazitäten der labore, (---)07 und es liegt natürlich auch an den (.)08 überMITTlungskapazitäten durch die gesundheitsämter; (--)


Eine in vergleichbarer Weise exponierte Relativierung der Daten ist in den Pressekonferenzen der DGS nicht zu beobachten, wenngleich sich auch dort Erläuterungen zum Zustandekommen der Zahlen finden. So wird beispielsweise der extreme Anstieg der Sterbefälle in Seniorenheimen durch die Zunahme der meldenden Einrichtungen erklärt (vgl. Ausschnitt 10) oder die Datenquelle (*SOS Médecins*, vgl. Ausschnitt 11) wird – informationsstrukturell allerdings im Hintergrund – mitgenannt:

(10) DGS, 03.04.20, 06:04-06:14

01 de plus en plus d’établissements (--) font (-)   *immer mehr Einrichtungen machen*02 aujourd’hui des remontées systématiques,   *heute systematische Meldungen*03 **ce qui explique ce nombre** (-) **important**   *was diese hohe Zahl erklärt*04 **et fortement augmenté par rapport à celui d’hier**,   *die im Vergleich zur gestrigen stark angestiegenen ist*

(11) DGS, 03.04.20, 03:44-03:52

01 **SOS médecins-** (--)   *SOS Médecins*02 **nous fait remonter** 1038 interventions, (--) […]   *meldet uns 1038 Einsätze*


Die Lagebeschreibung bleibt nicht auf eine reine Auflistung der Meldezahlen beschränkt. Die Angaben werden auch in Bezug zueinander gesetzt. Einerseits werden die Infektionszahlen verschiedener Länder einander gegenübergestellt und in der bildschematischen Vorstellung auf einer Skala angeordnet: »MEHR fälle gemeldet (‑) außerhalb chinas als IN china, (‑-)« (Beispiel 4), »le pays le plus touché en nombre absolu (.) après la chine (.) est l’italie (‑-) [...] suivent l’espagne […] et l’allemagne« (Beispiel 5). Andererseits werden die Meldezahlen zu verschiedenen Zeitpunkten miteinander verglichen: »das ist ’ne zunahme von knapp (.) ZEHN prozent gegenüber vorgestern (.)« (Beispiel 6), »aujourd’hui [...] par rapport à celui d’hier,« (Beispiel 10).^10^ Durch die Auf- und Abwärtsbewegung auf einer Skala erhält die Lagebeschreibung ein dynamisches Moment, sodass Entwicklungen nachvollzogen bzw. projiziert werden, die wiederum in den Figuren der kurve und der welle bildlich erfasst werden können (vgl. zur welle Klosa-Kückelhaus 2020a):

(12) DGS, 03.04.20, 22:40-22:44

01 […] nous ne sommes pas du tout encore, (--)      *wir sind noch lange nicht*02 **au sommet de cette** (.) **de cette VAGUE**, (-) […]   *am Höhepunkt dieser dieser Welle*

(13) RKI, 13.03.20, 12:55-13:05

01 wir wollen versuchen, (--)02 mit allen uns zur verfügung stehenden mitteln (---)03 **diese** (--) **KURve diese epidemische kurve** (.)04 **abzuflachen,** (.)05 **die** (--) **basis breiter zu machen** […]


In (12) wird das Pandemiegeschehen durch die Metapher der welle als dynamisches Phänomen konzeptualisiert, das qua Form einen Anstieg bis zum Scheitelpunkt beinhaltet, wodurch die weitere Zunahme der Infektionen – gleichzeitig aber auch ein irgendwann einsetzender Rückgang – prognostizierbar wird. Durch diese Konzeptualisierung werden die zunächst in Form einer inventarisierenden Liste erfassten Kategorien und Zahlen in »dramatisierbare Verläufe« (Contzen/Griem 2020, S. 244) eingebettet. Wie Ausschnitt 13 zeigt, werden zuweilen auch die in den Lageberichten formulierten Ziele und Maßnahmen direkt auf die Manipulation dieser Figuren bezogen (»diese epidemische kurve (.) abzuflachen (.) die (‑-) basis breiter zu machen«, Beispiel 13, vgl. auch die im weiteren Verlauf der Pandemie verbreiteten Losungen *flatten the curve, die Welle brechen*). Contzen/Griem (2020, S. 243) sprechen in diesem Zusammenhang von den »Affordanzen« der Form: Die Dinge teilen uns mit, was wir mit ihnen tun sollen.

### »nous sommes face à une épidémie nationale qui progresse vite« (DGS, 17.03.20) – Situationsdeutung und Lagebewertung

Wie die von den Beteiligten an den Pressekonferenzen des RKI verwendete Bezeichnung *Lageeinschätzung* erwarten lässt, geht mit der Darstellung auch eine Deutung und Bewertung der Situation einher.^11^ So werden beispielsweise die Meldezahlen in Ausschnitt 4 als Evidenzen für die Einordnung der globalen Situation als Pandemie präsentiert (»das zeigt also (das/dass) äh (‑) was wir hier wissen, (.) es handelt sich um eine pandemie,«).

Die nationale Lage in Deutschland wird in den Pressekonferenzen des RKI wiederholt – und für Lai:innen sehr abstrakt – als »dynamisch« charakterisiert. In Beispiel 14 wird das Verhältnis der aktuellen Meldezahlen zu den Zahlen des Vortags (»1174 fälle mehr«) mit »also ’ne starke dynamik;« reformuliert und damit gedeutet:

(14) RKI, 17.03.20, 05:59-06:11

01 das heißt also wenn wir (.) das verGLEIchen (-)02 ähm die zahlen (--) f/ 10 15 uhr gestern (.)03 der stand 6200 gegenüber vorgestern,04 dann haben wir **1174 fälle mehr.** (.)05 **also ‘ne starke dynamik**; […]


Die aus den Fallzahlen abgeleitete *Dynamik* wiederum wird – im folgenden Beispiel explizit – als Grund für eine Hochstufung des Gesundheitsrisikos angeführt (Z. 04-10):

(15) RKI, 17.03.20, 07:48-08:25

01 noch ein wort ähm (1.0)02 zur (---) lageeinschätzung. (2.1)03 wir haben (--) heute, (-)04 oder wir WERden heute die risikoeinschätzung (---)05 für die gesundheit der bevölkerung06 in deutschland ändern. (1.5)07 **wir werden sie** (-) **ab heute** (--)08 **als HOCH einschätzen.** (1.7)09 **der grund,** (-) **ist ganz einfach.** (.)10 **sie äh sehen die dynamik,** (---)11 ähm (-) sie (-) wir haben unter anderem gestern bereits:12 hat professor schaade ihnen auch vermittelt dass es (-)13 eine reihe von hinweisen14 von lokalen gesundheitsämtern gibt (-)15 °h die von einer sehr dynamischen lage sprechen, (.)


Bemerkenswert ist in Beispiel 15, dass »lageeinschätzung« (Z. 02) kurz darauf durch »risikoeinschätzung« (Z. 04) substituiert wird. Damit stellt Wieler seine Einschätzung potentiell in den Kontext einer modernen Risikopolitik, in der Ereignisse wie die Pandemie als (wissenschaftlich) berechenbare sowie gesellschaftlich beeinflussbare und regulierbare Risiken (und nicht etwa als »blinde[s] Schicksal«) betrachtet werden (vgl. Reckwitz 2020, S. 242 ff.; in Anlehnung an Luhmann 1991). In diesem Sinne nimmt die Verwendung des Risikobegriffs hier bereits die Notwendigkeit des Eingreifens und der (staatlichen) Regulierung zur Risikovermeidung vorweg (siehe dazu 3.3).

Auch in den Pressekonferenzen der DGS werden Situationsdeutungen vorgenommen, die durch Daten legitimiert bzw. aus diesen abgeleitet werden. Teilweise sehr explizit geschieht dies in den Ausschnitten 16 und 17, die im Zusammenhang zu betrachten sind:

(16) DGS, 17.03.20, 01:47-02:03

01 en france nous sommes face à une épidémie (-) nationale,   *in Frankreich stehen wir vor einer nationalen Epidemie*02 qui progresse (.) vite, (-)   *die schnell voranschreitet*03 °h avec des zones de circulation virale (.)      *mit Gebieten von*04 rapide et intense, (-)   *schneller und intensiver Viruszirkulation*05 aujourd’hui nous avons 7730 cas confirmés (.)   *heute haben wir 7730 Fälle bestätigt*06 par test pcr, (-)   *durch PCR-Test*


Es schließen sich weitere Zahlen zu folgenden Kategorien an: Hospitalisierungen, Genesene und sich in häuslicher Quarantäne befindende Personen, schwere Fälle auf Intensivstationen, aus dem Krankenhaus entlassene Personen. Der Sprecher gibt an, dass sich die Fallzahlen alle drei Tage verdoppelten und alle Regionen Frankreichs und der Überseegebiete betroffen seien. Die sechs am meisten betroffenen Regionen werden aufgezählt. Schwere Verläufe würden auch bei Erwachsenen unter 60 Jahren beobachtet, von denen einige auf den Intensivstationen betreut würden. Man beklage 175 Todesfälle, darunter sieben Prozent unter 60 Jahren.

(17) DGS, 17.03.20, 03:16-03:23

01 nous sommes donc en stade 3 de cette épidémie de (.)   *wir befinden uns also im Stadium 3 dieser Epidemie*02 covid-19,   *Covid-19*03 nous évoluons (-) progressivement vers une épidémie (.)   *wir bewegen uns nach und nach auf eine Epidemie zu*04 généralisée sur le territoire, (-)   *die sich auf dem gesamten Staatsgebiet ausbreitet*


Ausschnitt 16 beginnt mit einer Lageeinschätzung des Sprechers: Frankreich stehe einer schnell fortschreitenden nationalen Epidemie mit Zonen schneller und intensiver Zirkulation des Virus gegenüber (»face à«). Die folgenden – hier aus Platzgründen zusammengefassten – Daten können als Belege für diese Deutung verstanden werden. In Ausschnitt 17, der kurz danach anschließt, formuliert der Sprecher erneut eine Situationsdeutung, die mithilfe des Konnektors *donc *dieses Mal explizit als (erwartbare) Schlussfolgerung aus den genannten Daten präsentiert wird.^12^ Die epidemiologische Lage wird als »stade 3« klassifiziert, womit sich der Sprecher stillschweigend auf den nationalen Pandemieplan bezieht: »stade 3« ist dort definiert durch schnell steigende Fallzahlen, die den Anfang einer epidemischen Welle indizieren. Ab diesem Stadium sieht der Plan Kontaktbeschränkungen zur Verlangsamung der Ausbreitung des Virus und zur Vorbeugung gegen eine Überlastung des Gesundheitssystems vor (vgl. Ausschnitt 21).^13^ Diese intertextuelle Referenz bleibt allerdings implizit und die Nachvollziehbarkeit der Schlussregel setzt beim Publikum eine entsprechende Kenntnis der Quelle voraus. Des Weiteren heißt es: »nous évoluons (‑) progressivement vers une épidémie (.) généralisée sur le territoire, (‑)« (Z. 03 f.). Wie schon am Beginn des Ausschnitts 16 drückt der Sprecher seine Situationsdeutung hier mithilfe bildschematischer Metaphern aus. Die Epidemie wird erfasst als etwas, dem die Bevölkerung Frankreichs *gegenüber*steht (»nous sommes face à«, Beispiel 16), das mit hoher Geschwindigkeit fortschreitet (»qui progresse (.) vite«, Beispiel 16) und auf das sich die Bevölkerung ihrerseits zubewegt (»nous évoluons (‑) progressivement vers [...]«, Beispiel 17). Die bildliche Repräsentation der Lage entspricht hier einer drohenden Kollision mit der sich nähernden Katastrophe. Der an anderer Stelle geäußerte Appell »faisons barrière (.) au virus«, (DGS, 17.03.20, 04:47-04:49, wörtlich: ›errichten wir eine Barriere gegen das Virus‹) fügt sich mit der Metapher der barrière (›Barriere‹/›Absperrung‹) kohärent in diese Konzeptualisierung ein. Nebenbei sei bemerkt, dass sich in verschiedenen Pressekonferenzen des RKI eine andere Verwendung der weg-Metapher findet (allerdings nicht am 17.03.20):

(18) RKI, 13.03.20, 19:02-19:11

01 und darum (-) können wir m:it ‘ner (.)02 ziemlich großen überzeugung sagen03 **dass wir am anfang dieser epidemie stehen,** (--)04 °h und darum (-) können wir diese maßnahmen05 alle noch in die wege leiten,


Anders als in der Darstellung Salomons, in der die Epidemie als etwas dargestellt wird, auf das sich Frankreich zubewegt, ist die Epidemie in Wielers Metaphorik selbst der Weg, an dessen Anfang die mit »wir« gemeinte Bevölkerung Deutschlands »steh[t]« (und auf dem sie entlang geht). Wieler nutzt dabei die Metapher geschickt zur Untermauerung seiner Einschätzung, dass Maßnahmen noch in die Wege geleitet werden könnten, (und legt implizit den Appell nahe, diese zu ergreifen bzw. zu unterstützen).^14^

Ein weiteres Merkmal der Lageeinschätzung in beiden Institutionen ist die Verwendung sprachlicher Intensivierungsverfahren, mit denen die Sprecher das Ausmaß der Epidemie, die Singularität und die Schwere der Lage hervorheben.^15^ Zu beobachten ist hier u.a. die Verwendung von Adjektiven wie dt. *erheblich *oder* intensiv *(im Beispiel zudem im Superlativ):

(19) RKI, 17.03.20, 01:49-01:57

01 zurzeit ist tatsächlich (-)02 europa im FOkus (.) dieser pandemie (--)03 hier haben wir die (--) ähm (-) **intensivste dynamik**. (---)


Im französischen Korpus wird die Lage wiederholt als *inédit *(›neuartig‹/›noch nie dagewesen‹) oder *exceptionnel *(›außergewöhnlich‹) charakterisiert, wobei die Bedeutung dieser Zuschreibung durch Kode-Adverbien^16^ wie *totalement *(›vollkommen‹) oder* absolument *(›absolut‹) intensiviert wird:

(20) DGS, 14.03.20, 13:50-13:55

01 […] un contexte **totalement inédit,** (--)      *ein völlig neuartiger Kontext*02 et très évolusif (-) **très évolutif.** (---)   *und sehr »évolusif« [Versprecher] sehr entwicklungsstark*


### »das ist der grund warum einfach noch stärkere maßnahmen notwendig geworden sind« (RKI, 17.03.20) – Handlungslegitimierung

Wie der weitere Verlauf der Pressekonferenzen zeigt, werden die Situationsdeutungen von den Sprechern unmittelbar als Argumente zur Legitimierung von Maßnahmen herangezogen. Die beiden folgenden Ausschnitte sind direkte Fortsetzungen der Beispiele 15 bzw. 16/17, die zur besseren Lesbarkeit hier in Teilen wiederholt werden:

(21) DGS, 17.03.20, 03:16-03:52

01 nous sommes donc en stade 3 de cette épidémie de (.)   *wir befinden uns also im Stadium 3 dieser Epidemie*02 covid-19,   *Covid-19*03 nous évoluons (-) progressivement vers une épidémie (.)   *wir bewegen uns nach und nach auf eine Epidemie zu*04 généralisée sur le territoire, (-)   *die sich auf dem gesamten Staatsgebiet ausbreitet*05 °h nous passons **logiquement** (---)      *wir wechseln logischerweise*06 d’une stratégie de détection (--)   *von einer Strategie der Fallfindung*07 de recherche systématique des cas (--)   *der systematischen Suche nach Fällen*08 de lutte contre la diffusion, (.)   *der Bekämpfung der Ausbreitung*09 à une stratégie (--) d’action collective,   *zu einer Strategie des kollektiven Handelns*10 qui s’appuie sur des mesures de confinement strictes, (--)   *die sich auf strenge Lockdown-Maßnahmen stützt*11 de distanciation sociale, (--)   *auf soziale Distanzierung*12 et de mise en œuvre (.) des mesures barrière (.)   *und auf Umsetzung von Maßnahmen zur Kontaktbeschränkung*13 par TOUT le monde, (-)   *durch alle*14 tout au long de la journée. (.)   *den ganzen Tag über*


Der von der Regierung vorgenommene Wechsel von der Strategie der Nachverfolgung von Einzelfällen zur Strategie des *confinement* wird vom Sprecher hier als »logische« Folge der Einstufung der Lage in das Stadium 3 des nationalen Pandemieplans dargestellt und damit legitimiert. An der sprachlichen Oberfläche wird die Rationalität der Schlussfolgerung durch das Modaladverb »logiquement« markiert, wobei auch hier wieder gilt, dass die Identifizierung der Schlussregel spezifisches Wissen erfordert.

Auch beim RKI werden die Maßnahmen mit der Situationsdeutung begründet:

(22) RKI, 17.03.20, 08:18-08:36

01 […] dass es (-) eine reihe von hinweisen02 von lokalen gesundheitsämtern gibt, (-)03 °h die von einer sehr dynamischen lage sprechen, (.)04 °hh das heißt also (-)05 **das ist der grund warum einfach** (--)06 **noch stärkere maßnahmen notwendig geworden sind** (-)07 °h **um** die verbreitung des virus weiter **zu** verlangsamen,08 und die behandlungskapazitäten nicht **zu** überfordern; […]


Das Argumentationsmuster wird dabei explizit an der sprachlichen Oberfläche indiziert: Mit »das ist der grund warum« wird die Notwendigkeit stärkerer Maßnahmen als Folge der Einschätzung der Lage als »dynamisch« präsentiert. Als weitere Legitimierungsressource werden zudem die mithilfe der *um...zu-*Konstruktion versprachlichten Zwecke der Maßnahmen herangezogen (»um die verbreitung des virus weiter zu verlangsamen, und die behandlungskapazitäten nicht zu überfordern«).

### »c’est maintenant qu’il faut changer de comportement« (DGS, 14.03.20) – Appelle und Handlungsanweisungen

Schließlich richten die Sprecher in ihren Lageberichten Appelle in Form von Aufrufen, Bitten, Forderungen, Empfehlungen, Ratschlägen usw. an das Publikum, das zur Mitwirkung an der Umsetzung der Maßnahmen aufgefordert wird:

(23) DGS, 17.03.20, 03:51-04:05

01 nous devons faire bouclier de la nation (-)   *wir müssen einen Schutzschild der Nation errichten*02 pour nos personnes âgées (.)   *für unsere alten Menschen*03 et les personnes les plus fragiles, (-)    *und die schwächsten Menschen*04 °h **nous faisons donc un appel**      *wir appellieren daher*05 à la solidarité nationale entre les générations,   *an die nationale Solidarität zwischen den Generationen*06 soyons civiques (--) devant ce contexte totalement inédit,   *lasst uns Gemeinsinn zeigen angesichts dieser völlig*07 et très évolutif. (---)   *neuartigen und sich sehr schnell entwickelnden Situation*

(24) RKI, 17.03.20, 11:05-11:23

01 ohne diese maßnahmen (-) müssten wir davon ausgehen02 dass wir in wenigen monaten,03 vielleicht mehrere millionen (--)04 krankheitsfälle haben. (--)05 und DAS wollen wir vermeiden,06 und darum möchte ich alle (-) **bitten**07 dass sie diese maßnahmen ernst nehmen, (-)08 und auch umsetzen, (-)09 sowie wir das ja seit vielen wochen (--)10 wirklich **FORdern.** (.)11 von (.) allen […]


Außer in den pauschalen Appellen zur Solidarität und Mitwirkung zeigt sich der appellative Charakter der Pressekonferenzen in einer Reihe von teils performativ formulierten Forderungen (vgl. Auszug 27) sowie in Ratschlägen und Empfehlungen (»quelques conseils de bon sens«, DGS, 14.03.20, 12:59-13:01, ›ein paar Ratschläge aus gesundem Menschenverstand‹; »das heißt also (‑) es gibt klare empfehlungen von uns«, RKI, 17.03.20, 16:30-16:32). Die Appelle werden von den Sprechern legitimiert und argumentativ gestützt, indem etwa moralische Prinzipien wie der Schutz vulnerabler Personen als Argumente herangezogen werden (in Ausschnitt 23: »pour nos personnes âgées (.) et les personnes les plus fragiles,«, Z. 02 f.) oder die unerwünschten Konsequenzen von Handlungsunterlassungen angeführt werden (Ausschnitt 24, Z. 01-04). Der Zusammenhang zwischen Appell und Begründung wird jeweils durch Konnektoren wie *donc *bzw. *also* oder *darum* (»nous faisons donc un appel«, »darum möchte ich alle bitten, (‑) dass«) explizit hergestellt.

Inhaltlich beziehen sich die in Form von Imperativen, unpersönlichen Konstruktionen oder mithilfe von Modalverben geäußerten Aufforderungen in der Anfangsphase der Pandemie teilweise auf sehr konkrete Verhaltensweisen, die zu unternehmen oder zu unterlassen seien (»sorgen sie dafür dass wenn sie erkrankt sind sie (.) in ihre armbeuge niesen«, RKI, 17.03.20, 19:03-19:07; »puisque je rappelle, (‑-) que si vous avez des symptômes bénins, (‑) il faut APpeler (.) votre médecin traitant«, DGS, 17.03.20, 07:47-07:52 ›denn ich erinnere daran, dass Sie bei milden Symptomen Ihren Arzt anrufen müssen‹). Im folgenden Ausschnitt äußert der Sprecher einen ganzen Katalog von erlaubten und untersagten Alltagshandlungen:

(25) DGS, 17.03.20, 06:09-06:41

01 **on peut** faire des actes (-) importants,   *man kann wichtige Angelegenheiten erledigen*02 **on peut** aller donner son sang, (--)   *man kann Blut spenden gehen*03 et c’est très important en ce moment, (.)   und das ist sehr wichtig im Moment04 °h **on peut** évidemment se faire suivre; (--)      *man kann sich natürlich behandeln lassen*05 mais **on peut** aussi (--) avoir des actions solidaires, (.)   *aber man kann sich auch solidarisch zeigen*06 téléphoner à des proches, (.)   *Verwandte anrufen*07 écrire, (-)   *schreiben*08 prendre des nouvelles, (-)   *sich nach ihnen erkundigen*09 voire faire des courses (.)   *sogar einkaufen gehen*10 pour les personnes les plus (-) vulnérables. (--)   *für die am meisten schutzbedürftigen Menschen*11 **par contre** (.)   *dagegen*12 **on ne peut pas** recevoir chez soi dans son foyer, (.)   *kann man nicht Gäste in seinem Haus empfangen*13 être invité ailleurs, (.)   *anderswo eingeladen werden*14 organiser des fêtes, (-)   *Partys organisieren*15 un anniversaire, (.)   *einen Geburtstag*16 un mariage, (-)   *eine Hochzeit*17 se rassembler avec des amis, (.)   *sich mit Freunden treffen*18 sortir sans raison, (-)   *ohne Grund nach draußen gehen*19 °h ou organiser des activités (.) collectives. (---)      *oder gemeinsame Aktivitäten organisieren*


Ein Unterschied zwischen den in den Pressekonferenzen des RKI und der DGS geäußerten Appellen ist allerdings bezüglich der Adressierung zu beobachten. Während Wielers Appell sich nicht nur an die Bevölkerung, sondern auch an die Einrichtungen des Gesundheitssystems und der kommunalen Verwaltung richtet (vgl. Beispiele 26 und 27), präsentiert Salomon sich als Vertreter einer Exekutive, die diese Art von Maßnahmen selbst ergreift bzw. durchsetzt (Beispiel 28):

(26) RKI, 17.03.20, 13:21-13:29

01 darum auch **nochmal mein wiederholter appell,** (---)02 **an alle landrätinnen,** (--)03 **landräte,** (.)04 **bürgermeisterinnen,**05 **bürgermeister;** (-)06 das sind ja diejenigen (-)07 die in deutschland darüber entSCHEIden (--)08 wie vor ort (-) mit patienten (.)09 mit den gesundheitsämtern umgegangen wird.

(27) RKI, 17.03.20, 12:12-12:19

01 und darum (-) äh **fordern wir** seit wochen, (--)02 **die krankenhäuser auf** (.)03 dass sie ihre (---) kapazitäten hochfahren, (.) […]

(28) DGS, 14.03.20, 09:47-10:04

01 nous avons mobilisé aussi (---)   *wir haben auch mobilisiert*02 tous les établissements médico-sociaux,   *alle medizinisch-sozialen Einrichtungen*03 qui sont en plan bleu (.) au niveau national, (-)   *die sich auf nationaler Ebene im Plan Bleu befinden*04 °h **nous avons mobilisé** les réanimateurs (-)      *wir haben die Intensivmediziner mobilisiert*05 et l’ensemble des équipes de réanimation (.)   *und alle Intensivpflegeteams*06 pour anticiper (--) l’arrivée en grand nombre (-)   *um vorbereitet zu sein auf das Auftreten großer Zahlen*07 de cas graves, (-)   *von schweren Fällen*08 et **nous avons mobilisé** (.) au plan national (.)   *und wir haben auf nationaler Ebene*09 la réserve sanitaire (-) […]   *die Gesundheitsreserve mobilisiert*


Hier zeigt sich eine abweichende Rollenkonstitution der beiden Akteure, die im engen Zusammenhang mit den institutionellen Funktionen von RKI als der die Bundesregierung beratenden Einrichtung auf der einen Seite und DGS als ausführender Abteilung des französischen Gesundheitsministeriums auf der anderen Seite zu sehen ist.

Ähnlich wie bei der Lageeinschätzung spielen auch bei der Versprachlichung der Appelle Intensivierungsverfahren eine Rolle. Intensiviert wird u.a. die Bedeutung von Verben, die die in der Lage gebotenen Handlungen ausdrücken (»die kapazität, (‑-) der beatmungsplätze (‑-) in deutschland *maximal *erhöhen. (1.6)«, RKI, 18.03.20, 07:49-07:59; »äh diese krise die auf uns zukommt (‑-) können wir (‑-) *optimal* managen«, RKI, 13.03.20; 19:33-19:37; »rester chez eux (.) *le PLUS possible*«, DGS, 17.03.20, 04:22-04:24, ›*so viel wie möglich* zu Hause bleiben‹; »réduire *au minimum* les contacts et les déplacements sur l’ensemble du territoire«, DGS, 17.03.20, 04:05-04:17, ›Kontakte und Reisen auf dem ganzen Staatsgebiet *auf ein Minimum* reduzieren‹).

Im Zusammenhang mit deontischen Äußerungen wird die Bedeutung von Modalverben oder unpersönlichen Konstruktionen durch Assertions- oder Kode-Adverbien (vgl. Weinrich 1982, S. 436 f.) intensiviert (»les mesures barrière (.) doivent *vraiment* (‑) être acquises TRÈS vite (.) par l’ensemble de la population (‑--)«, DGS, 14.03.20, 12:13-12:18, ›die Kontaktbeschränkungen müssen *wirklich *sehr schnell von der gesamten Bevölkerung angenommen werden‹; »il est donc (‑) *absolument* impératif (‑) d’être (.) TRÈS attentif (.) aux consignes officielles,«, DGS, 14.03.20, 14:04-14:09, ›es ist daher *absolut* erforderlich, die offiziellen Anweisungen genau zu beachten‹).

Nicht ausschließlich, aber vor allem im deutschen Korpus beobachten wir Intensivierungen des Sprechakts selbst, etwa durch die Verwendung von *verba dicendi* (»aber ich möchte auch nochmal sagen dass natürlich die bürgerinnen und bürger, (‑-) äh vieles dazu tun können (‑)«, RKI, 17.03.20, 18:46-18:50) und/oder durch Wiederholung (vgl. dazu auch Beispiel 26).

(29) RKI, 17.03.20, 09:58-10:04

01 […] weil **nochmal**,(--)02 wir müssen (.) alles tun (-) um JEde (-)03 mögliche infektionskette zu unterbrechen (-) […]^17^


Durch Intensivierungsverfahren hervorgehoben wird schließlich auch die Dringlichkeit der Appelle, indem die temporaldeiktischen Ausdrücke *maintenant *bzw. *jetzt *durch die syntaktische *mise en relief *im Französischen bzw. die prosodische Hervorhebung und Wiederholung im Deutschen fokussiert werden:

(30) DGS, 14.03.20, 09:20-09:23

01 **c’est maintenant** (.)   *es ist jetzt*02 **qu**’il faut changer de comportement. (---)   *dass wir unser Verhalten ändern müssen*

(31) RKI, 17.03.20, 13:54-14:12

01 auch die kliniken; (-)02 die bislang noch nicht so stark betroffenen sind;03 müssen **JETZT** (-) **jetzt** (-) **in diesem moment** (--)04 so wie es am wochenende auch gefordert wurde05 von minister spahn, (-)06 **JETZT** sich darauf vorbereiten07 ihre elektiv (--) äh operationen runterzufahren,08 und für die intensivbetten hochzufahren. (1.6)


### Die Lageberichte als Krisenkommunikation: Zusammenfassung

Ausgangspunkt der Lageberichte als abgrenzbare Bestandteile der Pressekonferenzen von DGS und RKI sind die »Daten zur Lage«. Gemeint sind damit die Meldezahlen der Covid-19-Infizierten sowie weitere Angaben zu Todesfällen, Hospitalisierungen, beatmungspflichtigen Patienten auf Intensivstationen etc. Dabei wird das Pandemiegeschehen – in beiden Institutionen in sehr ähnlicher Weise – durch die Kartographierung und die Selektion bestimmter Kategorien in ein Ordnungssystem eingefügt, durch das es – man könnte hinzufügen: als Risiko – messbar und damit bewertbar wird. Das über die Lage vermittelte Wissen wird mithilfe verschiedener Metaphern konstituiert und veranschaulicht. Länder und Regionen werden als container präsentiert, in denen das Virus zirkuliert (z.B. »IN china«, »außerhalb chinas«). Die damit verbundene Fokussierung nationaler Territorien steht in gewissem Widerspruch zur tatsächlichen Vernetzung der Welt und der globalen Ausbreitung des Virus, legt aber die Möglichkeit nahe, die Pandemie auf nationaler Ebene zu verhandeln und zu bekämpfen.^18^ Entwicklungen im Pandemiegeschehen werden u.a. durch die Metaphern der welle und des weges veranschaulicht bzw. durch die Metapher vorweggenommen (»nous ne sommes pas du tout encore, (‑-) au sommet de cette (.) de cette VAGUE, (‑)«, Beispiel 12) oder perspektiviert (»nous sommes face à une épidémie (‑) nationale«, Beispiel 16, vs. »dass wir am anfang dieser epidemie stehen«, Beispiel 18).

Die so präsentierten Daten zur Lage werden von den Akteuren in Argumentationen eingebunden, die auf die Legitimation politischer Maßnahmen und Appelle zielen und in vielen Aspekten den von Klein (2000) beschriebenen topischen Mustern politischen Rechtfertigungshandelns entsprechen. Diese umfassen im Wesentlichen die Handlungskategorien Situationsdarstellung, Situationsbewertung, Zielsetzung, Handlungsanforderung und Norm/Wert, die zueinander in einem Fundierungsverhältnis stehen, indem die Handlung einer Kategorie argumentativ durch die Handlung einer anderen Kategorie gestützt wird (vgl. Klein 2000, S. 628 ff.; siehe dazu auch den Beitrag von Spieß in diesem Heft). So werden Infektionszahlen und andere Messwerte in den Lageberichten als Begründungen für die Einschätzung der Lage als »dynamisch« bzw. »évolutif« oder des Risikos als »hoch« herangezogen (Motivationstopos). Hohe absolute Zahlen, im internationalen Vergleich relativ hohe Zahlen sowie die Zunahme von Fallzahlen über einen Zeitraum hinweg werden als Indizien einer kritischen Lage behandelt. An der sprachlichen Oberfläche signalisieren kausale oder konsekutive Konnektoren (z.B. fr. *donc*, dt. *also, darum*) entsprechende Grund-Folge-Relationen. Die Singularität und das Ausmaß der Pandemie werden durch Intensivierungsverfahren unterstrichen. Der konkrete Zusammenhang zwischen Messwerten und Lageeinschätzungen, wie er in epidemiologischen Modellrechnungen etabliert wird, dürfte für das Laien-Publikum allerdings opak bleiben. Hier manifestiert sich ein grundlegendes und häufig kritisiertes Dilemma der Krisenkommunikation in der Pandemie (vgl. u.a. Kuck 2020, S. 248 ff.; Nassehi 2020, S. 158 ff.; Vogel 2020). Mit der Nennung sehr detaillierter Daten und »szientoider Maßzahlen« (Nassehi 2020, S. 158) wird eine (vermeintliche) Transparenz und »behauptete gemessene Objektivität« (Kuck 2020, S. 250) der wissenschaftlichen Grundlage politischer Entscheidungen signalisiert, ohne dass die Darstellung die tatsächliche Komplexität epidemiologischer Modellierung und politischer Entscheidungsprozesse jedoch abzubilden vermag (vgl. auch Moirand 2021). Das Dilemma wird vor allem in den Pressekonferenzen des RKI deutlich, in denen die genannten Zahlen immer wieder relativiert und in einen erläuternden Kontext gestellt werden. Dieses Bemühen um Transparenz stößt aber an Grenzen, so dass letztendlich nur das Vertrauen in die Expertise der Akteure bleibt:

(32) RKI, 17.03.20, 00:27-00:40

01 und wir sind uns auch sehr (-) bewusst darüber02 dass es eine dunkelziffer gibt, (.)03 das alles (--) sagen wir (-) immer schon (.)04 aber es ist nicht immer so einfach für jeden (--)05 zu verstehen. (.)06 glauben sie dass wir (--)07 dieses business beherrschen. (--)


Die aus den Daten abgeleiteten Situationsdeutungen und -bewertungen dienen schließlich der Legitimierung bestimmter Maßnahmen, die darauf abzielen, die Ausbreitung des Virus zu verlangsamen, um die Kapazitäten des Gesundheitssystems nicht zu überlasten (Finaltopos), wobei auch gesellschaftliche Werte wie Solidarität und soziale Normen wie der Schutz vulnerabler Gruppen (Prinzipientopos), der Verweis auf den nationalen Pandemieplan (Autoritätstopos im weiteren Sinne) oder auch unerwünschte Effekte, die bei Unterlassung von Maßnahmen drohen (Konsequenztopos), als stützende Argumente herangezogen werden. Auch hier spielen Intensivierungen eine Rolle, indem sie den Ausdruck der Dringlichkeit und Umfänglichkeit der Maßnahmen verstärken. Die Lageeinschätzungen münden in den Appell zur Mitwirkung, wobei die Akteure in der ersten Phase der Pandemie der Zuhörerschaft praktisches Handlungswissen in Bezug auf Hygienemaßnahmen oder das Verhalten im *confinement* vermitteln. Anders als Salomon, der als Leiter der DGS Teil des französischen Gesundheitsministeriums ist und selbst Maßnahmen im Bereich des staatlichen Gesundheitswesens durchsetzen kann, muss der Präsident des RKI um politische Unterstützung durch kommunale Verwaltungsinstitutionen und Einrichtungen des Gesundheitswesens werben.

Auch wenn in den Pressekonferenzen durchaus weitere Aspekte der Pandemie vermittelt werden,^19^ bilden die Lageberichte mit der Präsentation der Fallzahlen und den aus ihnen abgeleiteten, immer wieder ähnlichen und von Tag zu Tag manchmal wortgleichen Situationsdeutungen, Handlungslegitimierungen und Appellen eine Konstante der Pressekonferenzen, die uns seit Anfang 2020 begleiten. Die Serialität verleiht den Berichten einen rituellen Charakter und trägt – zusammen mit den vielfältigen Möglichkeiten der öffentlichen und privaten Anschlusskommunikation – zur kollektiven Aufrechterhaltung des Krisenmodus bei.

## Nominationen

Eine besonders breite Wahrnehmung in der Sprache der Corona-Krise erfährt der Wortschatz, insbesondere Lexeme, die neuartig sind oder zumindest neuartig erscheinen. So sammelt das Leibniz-Institut für Deutsche Sprache in einem Neologismenverzeichnis sowohl tatsächlich neu entstandene Wörter als auch Ausdrücke, die im Rahmen der Pandemie Bedeutungsveränderungen erfahren haben (Leibniz-Institut für Deutsche Sprache 2021).^20^ Auf französischer Seite ist etwa die Rubrik »Les mots du virus« der Bibliotheken der Université d’Aix-Marseille zu nennen (Bibliothèques de l’Université d’Aix-Marseille 2021). Das folgende Kapitel vergleicht die Verwendung von lexikalischen Ausdrücken in den deutschen und französischen Lageberichten. Dabei geht es um die Frage, wie Fachterminologie vermittelt und neue Begrifflichkeiten eingeführt werden. Schwerpunktmäßig werden in einem ersten Teil die Bezeichnungen des Virus und im Anschluss etwas ausführlicher die Nominationen im Zusammenhang mit Masken analysiert.

### »die haben wir zurzeit für corona nicht« (RKI, 13.03.20) – Die Bezeichnung des Virus und der Krankheit

Im Deutschen wie im Französischen ist umgangssprachlich die Bezeichnung *Coronavirus* bzw. *coronavirus* am weitesten verbreitet. Da hiermit eigentlich eine Gattung von Viren bezeichnet wird, besteht im fachsprachlichen Diskurs die Notwendigkeit zur genaueren Benennung des neuartigen Virus mit der Bezeichnung *SARS-CoV‑2*:

(33) RKI, 03.04.20, 09:41-09:46

01 das kann eine übertragung, (-)02 von **sars-cov-2** (---)03 begünstigen. (---)


Dabei verschwimmt insbesondere in den französischen Lageberichten die Unterscheidung zwischen dem Virus und der Krankheit:

(34) DGS, 30.04.20, 03:26-03:33

((Olivier Véran, Gesundheitsminister:))01 si (.) c’est entre 6 et 10 pourcent.   *wenn zwischen 6 und 10 Prozent*02 des patients qui vont aux urgences; (-)   *der Patienten, die die Notaufnahme aufsuchen*03 pour une **suspicion de coronavirus**, (-) […]   *bei einem Verdacht auf Coronavirus*


An dieser Stelle wäre die akronymische Bezeichnung der Krankheit (*Covid-19*) treffender gewesen. Anders als im Deutschen gibt es im Französischen keine umgangssprachliche Verkürzung des Ausdrucks *Coronavirus* zu *Corona*, was wiederum sowohl Virus als auch Krankheit bezeichnen kann, wie in folgendem Auszug^21^ sichtbar wird:

(35) RKI, 13.03.20, 45:44-45:50

01 gegen influenza gibt es ja impfstoffe. (--)02 das ist ja ‘ne ‘ne maßnahme gegen influenza, (.)03 die haben wir zurzeit für **corona** nicht. […]


Durch die Bezeichnungskonkurrenz von fachsprachlichen und umgangssprachlichen Termini kommt es zuweilen zu Reformulierungen. Im folgenden französischen Auszug wird zunächst die eigentlich ungenaue Benennung der Krankheit als Virus (»épidémie de coronavirus«) im Anschluss durch »covid-19« reformuliert:

(36) DGS, 24.02.20, 08:33-08:38

01 euh nous approchons désormais (.) les 80000 cas, (--)       *wir nähern uns jetzt den 80000 Fällen*02 pour cette **épidémie de coronavirus** (.)   *bei dieser Coronavirus-Epidemie*03 **covid-19**, (.) […]   *Covid-19*

(37) RKI, 03.04.20, 02:29-02:35

01 die fallzahlen werden täglich aktualisiert, (-)02 sie können sie (-) ganz DEtailliert, (--)03 auf unserem dashboard abrufen,04 das sie über unsere **corona** (-)05 **covid-19 website** finden können, (--)


Die Koexistenz beider Benennungsmodi *SARS-CoV‑2* vs. *Coronavirus* sowie *Covid-19* vs. *Corona *weist darauf hin, dass die Akteure der Lageberichte einerseits als Vertreter medizinischer Fachinstitutionen – dies gilt insbesondere für das RKI – für die Verwendung der präziseren Terminologie einstehen, was an den vorigen Beispielen dadurch deutlich wird, dass diese als reformulierendes Element erscheinen. Andererseits verwenden auch sie die medial verbreiteteren Ausdrücke *Corona(virus)*, wobei im weiteren Verlauf der Lageberichte zu untersuchen wäre, inwieweit sich die in der Frühphase auftretenden Konkurrenzen im Laufe der Zeit auflösen und sich die Benennung zugunsten eines Ausdrucks normalisiert.

### »das sind diese richtigen masken« (RKI, 14.04.20) – Die Bezeichnung von Masken

Masken stellen ein zentrales Element des Coronadiskurses dar. Daher sind sie auch ein wiederkehrender Topos in den Lageberichten sowohl des RKI als auch der DGS. Anders als bei der Benennung des Virus, wo es um Bezeichnungskonkurrenzen geht, spielen bei den Masken nicht nur Bezeichnungs-, sondern auch Bedeutungskonkurrenzen (vgl. Hermanns 2007, S. 202) eine entscheidende Rolle in der Entwicklung der Lexik. In beiden Institutionen erfährt die Maske als Maßnahme des Infektionsschutzes einen konzeptuellen Wandel, der sich in den Nominationen niederschlägt. Zunächst wird sowohl in Deutschland als auch in Frankreich die Maske ausschließlich als Teil des medizinischen Arbeitsschutzes betrachtet. Beim RKI besteht zunächst eine ablehnende Haltung gegenüber dem Alltagsgebrauch von Masken dahingehend, dass »keine evidenz« bestehe, dass ein Schutz durch das Tragen einer Maske gegeben sei:

(38) RKI, 13.03.20, 38:45-38:56

01 und es ist einfach so wenn sie draußen, (--)02 als äh mensch (.) mit einer maske rumlaufen03 suggerieren sie einen schutz davor04 aber es gibt keine evidenz dafür05 dass dieser schutz gegeben ist (1.3)


Später wird eine Chance darin gesehen, dass man sich durch eine Maske schützen könne, und im Juni 2020, der hier ausblickend nur kurz erwähnt sei, benutzt das RKI die auch politisch propagierte AHA-Regel, wonach man in zahlreichen Situationen eine Alltagsmaske zu tragen habe:

(39) RKI, 01.04.20, 49:58-50:06

01 °h und wenn man dann natürlich02 solchen **mund-nasen-schutz** trägt, (-)03 °h dann ist die chancë? (-)04 dass man weniger viren ausscheidet05 größer. davon

(40) RKI, 23.06.20, 08:19-08:35

01 äh deshalb ist es wirklich weiter so wichtig,02 dass wir uns alle, (.)03 nach wie vor achtsam verhalten,04 und uns an die (.) **aHA-regeln** halten also (-)05 abstand halten (--)06 hygieneregeln beachten °hh07 und dort wo geboten °h masken tragen08 alltagsmasken tragen. (1.4)


Bei der DGS ist der Wandel etwas pointierter: Am Anfang heißt es, man dürfe keine Masken tragen, was durch den Mangel an Masken zu begründen ist:^22^

(41) DGS, 17.03.20, 10:29-10:42

01 il ne faut pas mettre le masque (.)   *man sollte die Maske nicht aufsetzen*02 quand on n’est pas professionnel de santé,   *wenn man nicht im Gesundheitswesen tätig ist,*03 et il ne faut pas porter de gants, (-)   *und man sollte keine Handschuhe tragen*04 la meilleure solution est de se laver les mains (--)   *die beste Lösung ist sich die Hände zu waschen*05 régulièrement. (---)   *regelmäßig*06 donc ne portez pas des masques,   *also tragen Sie keine Masken*07 soyez solidaires °h avec nos soignants   *seien Sie solidarisch mit unseren Pflegekräften*08 qui se battent (.) au quotidien.   *die tagtäglich kämpfen*


Später im April wird eingeräumt, dass, wenn die Allgemeinheit es wolle (»s’il le souhaite«, Beispiel 42), es gewissermaßen freigestellt sei, ob man eine Maske trage oder nicht. Der weitere Kontext geht damit einher, dass Masken der asiatischen Kultur zugeschrieben werden und das Tragen von Masken in Frankreich nicht so selbstverständlich gegeben sei. Im Mai schließlich finden wir den Befehl, Masken in bestimmten Kontexten zu tragen:

(42) DGS, 03.04.20, 15:29-15:37

01 et donc si nous avons l’accès à des masques,   *und wenn wir also Zugang zu Masken haben*02 **nous encouragerons** effectivement (-) le: grand public   *werden wir tatsächlich die breite Öffentlichkeit ermutigen*03 **s’il le souhaite**,   *wenn sie es wünscht*04 à porter des masques,   *Masken zu tragen*05 en particulier **ces masques alternatifs**   *insbesondere diese alternativen Masken*06 qui sont en cours de production.   *die gerade hergestellt werden*

(43) DGS, 19.05.20, 15:20-15:32

01 **et portez systématiquement**, (.)   *und tragen Sie grundsätzlich*02 **un masque grand public** en privilégiant l’accès (-)   *eine Alltagsmaske und ermöglichen Sie regelmäßig Zugang*03 régulier aux solutions hydroalcooliques   *zu Desinfektionsmitteln*04 dans vos déplacements. (1.1)   *wenn Sie unterwegs sind*05 avec des visiteurs à la maison (.)   *mit Besuchern zu Hause*06 °hh **portez un masque en permanence** (.)       *tragen Sie ständig eine Maske*07 ainsi que votre visiteur, […]   *so wie auch Ihr Besucher*


Wenn man beide Institutionen im Hinblick auf die je drei Beispiele (38–40 und 41–43) anschaut, dann lassen sich hier leicht unterschiedliche deontische Kategorien zuweisen. Zunächst ist es beim RKI vor allem das Abraten und das Warnen vor dem vermeintlichen Schutz der Maske und der falschen Hoffnung in diese Maßnahme. Schließlich wird das Tragen zur Verpflichtung. Bei der DGS ist es erst das Verbot, dann die Freistellung – das Tragen einer Maske ist fakultativ – und schließlich ebenfalls die Verpflichtung.

Dass sich die Argumentation so stark wandelt, geht mit einer Veränderung des Konzepts von *Maske* einher: Bei der DGS wird der Wandel dadurch sehr offenkundig, dass Alltagsmasken ins Spiel kommen. Diese beseitigen das Problem des Mangels, da sie unbegrenzt produzierbar und jederzeit selbst herstellbar sind. Beim RKI ist die Idee der Alltagsmaske wohl schon früher vorhanden, allerdings mit der Skepsis behaftet, dass sie nicht ausreichend schütze. Deswegen geht vor allem beim RKI die konzeptuelle Aushandlung mit einer terminologischen Aushandlung von Masken einher. Am 14.04.2020 erfolgt eine Dreiteilung in Atemschutz-Masken – auch bezeichnet als »richtige masken« (RKI, 14.04.20, 47:25) –, OP-Masken und einer dritten Kategorie, die nicht als Mund-Nasen-Schutz verstanden werden soll:

(44) RKI, 14.04.20, 48:56-49:15

01 das DRITte worüber jetzt02 in den letzten wochen oft gesprochen wird,03 °hh das nennen wir äh mal (.)04 das nennen manche **community mask**,05 oder wir nennen es jetzt äh (-)06 **mund-nasen** (-) **bedeckung**. (1.0)07 warum sagen wir **nicht schutz**, **sondern bedeckung**. (.)08 erstens (-)09 °h ähm diese (.) selbstgemachten oder stoffmasken10 wie immer man die nennt11 diese selbstgemachten mund-nasen-bedeckungen, […]


Neben den gemeinsprachlichen Termini fr. *masque* und dt.* Maske* war auch vor der Corona-Pandemie im Deutschen das Wort *Mundschutz* gängig (vgl. Klosa-Kückelhaus 2020b). Dieser Ausdruck wird in den Pressebriefings des RKI jedoch kaum verwendet, wohl auch aus der Erfahrung heraus, dass Lai:innen die Nase beim Tragen oft nicht bedecken. Entsprechend ist viel stärker vom *Mund-Nasen-Schutz* die Rede, was vor der Corona-Pandemie noch kein gängiger Terminus war. Als Abgrenzung von Schutzmasken wird für die dritte Kategorie der Alltagsmasken explizit die Benennung als *Mund-Nasen-Bedeckung *eingeführt, es handelt sich also gewissermaßen um eine deontische Bedeutungskonkurrenz (vgl. Klein 2009, S. 2122), d.h. das gemeinsprachliche Konzept einer Maske als Instrument des Infektionsschutzes soll durch die fachlich motivierte Umbenennung des Experten korrigiert werden. Der alltagssprachliche Gebrauch des Ausdrucks *Mund-Nasen-Bedeckung* könnte weiter untersucht werden. Dass er klar abgrenzbar vom *Mund-Nasen-Schutz *verstanden wird, ist jedoch zu bezweifeln.

Auch in den Lageberichten der DGS ist die konzeptionelle Dreiteilung von Masken nachvollziehbar. So werden wie beim RKI die Termini *FFP2, FFP3* auch bei der DGS *masques P2, P3* der Allgemeinheit vermittelt, die vor der Pandemie wohl kaum damit vertraut war. Die zweite Kategorie wird gemeinhin als *masques chirurgicaux *bezeichnet. Interessant ist noch zu erwähnen, dass Salomon am 11.03.2020 noch in einer ablehnenden Haltung gesagt hat, dass (medizinische) Masken nicht »pour le grand public«, d.h. für die breite Bevölkerung, gedacht seien, während sich später gerade der Terminus *masques grand public *neben *masques alternatifs* für Alltagsmasken durchsetzt und auch offizielle Verwendung findet (vgl. Beispiel 42).

Die genannten Beispiele erweitert um eine Auswertung weiterer softwaregenerierter Transkripte der deutschen und französischen Lageberichte von Februar bis April 2020 ergeben die in Tabelle 1 zusammengestellten Bezeichnungen für Masken.

**Tab. 1:** Ausdrücke zur Bezeichnung von Masken

**Masken-typ**	**Favorisierte Termini (RKI)**	**Spontane oder alternative Bezeichnungen (RKI)**	**Favorisierte Termini (DGS)**	**Spontane oder alternative Bezeichnungen (DGS)**
1	FFP2-/FFP3-Masken	Richtige Masken	Masques P2/P3 Masques FFP2	Masques des professionnels Masques réservés aux soignants ≠ Masques pour le grand public
2	Mund-Nasen-Schutz	OP-Masken Chirurgische Masken	Masques chirurgicaux	
3	Mund-Nasen-Bedeckung Alltagsmasken	Mund-Nasen-Schutz *Community mask* Selbstgemachte Masken Stoffmasken Textilmasken Behelfsmasken	Masques alternatifs Masques grand public	Masques dits alternatifs D’usage on va dire quotidien

In dieser Übersicht wird nach den Termini getrennt, die etabliert sind bzw. im Diskurs etabliert werden sollten, und jenen, die spontan formuliert oder als (zunächst) weniger favorisierte Bezeichnungen verstanden werden müssen. Letzteres erkennt man etwa an modalisierenden Rahmungen wie im Beispiel 44: »*das nennen manche* community mask, *diese* (.) selbstgemachten oder stoffmasken«. Der Demonstrativbegleiter als Distanzsignal ist ebenso im Französischen feststellbar (vgl. Beispiel 42: »*ces* masques alternatifs«).

### Nominationen in den Lageberichten zwischen Fach- und Alltagsdiskurs – Zusammenfassung

Die Lageberichte können im Hinblick auf die Verwendung von Terminologie unter verschiedenen Prämissen verstanden werden: Als medizinische Fachinstitutionen werden die jeweiligen Vertreter als Wissenschaftler wahrgenommen, von denen zu erwarten ist, dass sie das epidemiologische Geschehen in präziser Fachterminologie ausdrücken. Dies ist etwa am Insistieren auf die Bezeichnung *Covid-19* in beiden Sprachen sichtbar. Sie müssen jedoch auch dem Laienpublikum, wo nötig, die Terminologie erklärend vermitteln (vgl. hierzu auch die Ausführungen zu den verschiedenen Zahlen in Abschnitt 3.1). In ihrer politischen Rolle als Instanzen der staatlichen Krisenkommunikation spielt auch der Versuch der Etablierung von Begrifflichkeiten (»mund-nasen (‑) bedeckung.«, Beispiel 44) sowie die Verbreitung gesundheitspolitisch eingeführter Ausdrücke eine Rolle (»aHA-regeln«, Beispiel 40). Weiterhin nehmen die Akteure der Lageberichte medial verbreitete Lexeme, die nicht im Fachdiskurs entstanden sind, im Sinne einer Experten-Laien-Interaktion auf und bearbeiten sie (»des (.) masques (‑-) *dits* (.) alternatifs. (‑-)«, DGS, 03.04.20, 14:47-14:49, ›*sogenannte* alternative Masken‹).

## Fazit

Die vergleichende Analyse der deutschen und französischen Lageberichte hat – bei aller Unterschiedlichkeit der Funktionen der beteiligten Akteur:innen und Institutionen – zahlreiche Ähnlichkeiten zwischen der Krisenkommunikation von DGS und RKI aufgezeigt. Diese zeichnet sich in beiden Fällen durch die Bereitstellung und Deutung von Daten aus, mithilfe derer staatliche Maßnahmen legitimiert bzw. Handlungsaufforderungen begründet werden, und weist typische Argumentationsmuster des politischen Rechtfertigungshandelns auf. Ein in der Analyse noch nicht systematisch berücksichtigter Aspekt der Krisenkonstitution ist die Serialität der Lageberichte, was insbesondere durch den einzelsprachlichen Vergleich mehrerer aufeinanderfolgender Berichte untersucht werden könnte. Durch das regelmäßig wiederkehrende – d.h. in der hier betrachteten Phase der Pandemie: fast tägliche – Vermelden der Messzahlen und ihre Einordnung auf einer auf- oder absteigenden Skala erhält das Geschehen eine ›in Echtzeit‹ nachvollziehbare Dynamik und wird der Krisenmodus aufrechterhalten.

Neben der Vermessung der Krise gehört zu den Lageberichten auch die Vermittlung von pandemierelevantem Alltagswissen, unter anderem über Hygiene- und Kontaktverhalten oder den Gebrauch von Masken. Vor allem an diesem Beispiel konnte gezeigt werden, dass die Wissenskonstitution und -vermittlung mit lexikalischen Aushandlungsprozessen einhergeht. Zu berücksichtigen sind dabei Rollenkonflikte zwischen Wissenschaft, Politikberatung und politischer Exekutive. Insgesamt kommt der Rollenkonflikt zwischen Wissenschaft und Politik beim RKI stärker zum Ausdruck, während die DGS deutlicher eine politische Stimme repräsentiert.

## Footnotes

1 Zur Vermittlung der Krise auf politischer Ebene vgl. etwa die romanistischen Analysen von Hesselbach (2020) und Leschzyk (2020) sowie deutsch-französisch kontrastiv Spieß (2020).

2 Mitte März 2020 bestand aus Sicht europäischer Virolog:innen und Epidemiolog:innen kein Zweifel mehr an der Gefahr des neuartigen Coronavirus. Zu den Abwägungen der Wochen zuvor vgl. mit besonderem Fokus auf das RKI das Kapitel »Covid-19: War die Katastrophe vorhersehbar?« in Mukerji/Mannino (2020, S. 23–62).

3 Eine detaillierte Beschreibung der Zuständigkeiten ist auf der Website des Instituts zu finden (Robert Koch-Institut 2020).

4 Die Transkription folgt weitgehend den Richtlinien von *GAT 2 *(Selting et al. 2009). Verzichtet wird auf Sprechersiglen, da es sich, sofern nicht anders gekennzeichnet, nur um Beiträge von Lothar Wieler bzw. Jérôme Salomon handelt. Bewusst von den gängigen Konventionen der Transkription abgewichen wurde bei der Verschriftlichung der Zahlen: Da Zahlen besonders häufig sind, werden zur besseren Lesbarkeit Ziffern verwendet, sofern die Prosodie nicht besonders hervorgehoben werden muss. Zu den französischen Textausschnitten wird eine, soweit möglich, interlineare Übersetzung ins Deutsche angeboten.

5 Folgende Pressekonferenzen wurden vollständig berücksichtigt: *Points de situation* vom 14.03.2020, 17.3.2020 und 03.04.2020 sowie die Pressebriefings des RKI vom 13.03.2020, 17.03.2020, 18.03.2020 und 03.04.2020. Hinzu kommen kürzere Ausschnitte weiterer Konferenzen aus dem ersten Halbjahr 2020.

6 Nebenbei sei angemerkt, dass die Zahlen des RKI und die der DGS an diesem Tag offensichtlich nicht übereinstimmen.

7 Auf das Vertextungsmuster der Beschreibung weist auch schon das metaphorisch verwendete visuelle Wahrnehmungsverb »schauen wir uns: (.) zunächst mal [...] an« (Auszug 4) hin (vgl. Hausendorf/Kesselheim 2008, S. 97 ff.).

8 Außersprachlich findet die hier zu beobachtende Fokussierung nationaler Territorien in dieser Phase der Pandemie eine Entsprechung in der Schließung von Grenzen, der Errichtung von Grenzzäunen oder der Rückholung von Reisenden auf das nationale Territorium (vgl. auch Knoblauch 2020, S. 8).

9 Teilweise werden auch Differenzierungen nach Alter und Geschlecht vorgenommen. Quantifiziert werden zudem die durchgeführten Tests, die Auslastung von Notaufnahmen und Intensivstationen sowie – in Frankreich – die Anzahl der Transfers von Erkrankten und Pflegepersonal in andere Regionen.

10 Moirand (2021) beobachtet ähnliche, von ihr als »anxiogène« (›angsteinflößend‹) beschriebene Vergleichsstrukturen in den Bildschirmlaufschriften des Nachrichtensenders BFMTV, in denen unablässig die aktuellen Zahlen angezeigt werden: »L’Espagne passe la barre des 2000 morts, 462 de plus en 24h«; »Déjà plus de morts en Italie qu’en Chine«.

11 Vgl. auch: »eine tägliche lageeinschätzung das ist unser (‑) job« (RKI, 03.04.20, 9:25-9:27).

12 Hansen (1997, S. 168) betrachtet *donc *als »marker of mutual manifestness«: *donc *signalisiert, dass der mit dem Konnektor markierte Sachverhalt den Beteiligten bekannt oder ihnen kognitiv zugänglich ist – z.B. weil der Sachverhalt bereits erwähnt oder allgemein bekannt ist oder weil er sich logisch/zwangsläufig aus dem Gesagten ergibt.

13 »L’augmentation rapide du nombre de cas signe le début de la vague épidémique. Le stade 3 marque l’arrêt de la surveillance individuelle des cas par l’institut de veille sanitaire. Il s’agit dès lors de limiter la contagion par des mesures barrières, de réduire la charge sur le système de santé, de limiter l’absentéisme au travail, de renforcer la capacité de réponse sanitaire.« (Gouvernement 2021).

14 Vgl. auch an anderer Stelle: »wir stehen am ANfang diese epidemie (1.3) wir sind einfach nur (‑) ein bis zwei wochen (‑) VOR italien. (‑) […] wir stehen am anfang, (.) und darum können wir doch die ganzen maßnahmen die wir die ganze zeit fordern, (‑--) die können wir ja umsetzen (‑)« (RKI, 18.03.20, 11:24-11:49).

15 Unter Intensivierungsverfahren verstehen wir sprachliche Mittel, die auf die Steigerung von Quantität oder Qualität des Gesagten auf semantischer Ebene, auf die Hervorhebung des Wahrheitsgehalts der Äußerung und/oder auf die Stärkung der illokutiven Kraft des Sprechakts zielen (Albelda/Briz 2021).

16 Zu Kode-Adverbien zählt Weinrich (1982, S. 468) Indefinit-Adverbien, die angeben, »daß die Text-Bedeutung eines Verbs, Adjektivs oder Adverbs das volle Maß dessen erreicht, was von der Kode-Bedeutung her überhaupt zugelassen ist. Ihr gemeinsames semantisches Merkmal ist <ganzheit>.«.

17 In diesem Beispiel spielt zudem die Verwendung von Indefinitpronomen und -begleitern (*alles, jede*) eine Rolle, die die Notwendigkeit allumfassender Maßnahmen unterstreichen. Ähnliches ist für *tous *im französischen Korpus zu beobachten: »et nous devons être TOUS (.) extrêmement civiques, (--)«, DGS, 14.03.20, 16:03-16:06, ›und wir müssen alle extrem gemeinsinnig sein‹.

18 Vgl. auch Bubenhofer et al. (2020, S. 163), die für die Deutschschweiz zeigen, dass sich der öffentliche Diskurs mit dem ersten Lockdown zunehmend auf die Probleme im Inland verengt.

19 Dazu gehören die Kapazität und die Einsatzweisen von Tests, generelle Informationen zu Krankheitsbild und Krankheitsverlauf sowie – besonders in der ersten Phase der Pandemie – die umstrittene Verwendung von Masken, auf die in Kapitel 4 noch näher eingegangen wird.

20 Zu weiteren deutschen Glossaren vgl. Möhrs (2020).

21 Dieses Beispiel gehört strenggenommen nicht zum Lagebericht des RKI, der als vorbereiteter Vortrag im ersten Teil der Pressekonferenzen stattfindet. Vielmehr handelt es sich um einen Auszug aus einer spontan formulierten Antwort auf eine Journalistenfrage.

22 Der Mangel an Masken kann als grundlegendes Motiv für die skeptische Haltung betrachtet werden, gleichwohl dieser Mangel seltener explizit erwähnt wird (z.B. am 17.03.20). Anstelle dessen werden eine ganze Reihe anderer Argumente gegeben, warum das Tragen von Masken nicht förderlich sei, die letztlich auf den Mangel zurückgeführt werden können: dass die Masken falsch getragen und falsch benutzt würden und dass ein zusätzliches Risiko bestehe, dass man sich häufiger ins Gesicht fasse: »donc on se contamine massivement en PENsant à être protégé«, DGS, 17.03.20, 10:26-10:29 ›also kontaminiert man sich massiv, während man sich geschützt glaubt‹.
